# The impact of COVID-19 pandemic on mental burden and quality of life in medical students – results of an online survey

**DOI:** 10.3205/zma001603

**Published:** 2023-04-17

**Authors:** Marie Halfmann, Lea Wetzel, Noah Castioni, Falk Kiefer, Sarah König, Astrid Schmieder, Anne Koopmann

**Affiliations:** 1 Central Institute of Mental Health (CIMH), Department of Addictive Behavior and Addiction Medicine, Mannheim, Germany; 2 University of Heidelberg, Feuerlein Centre on Translational Addiction Medicine (FCTS), Heidelberg, Germany; 3 University Hospital Würzburg, Institute for Medical Teaching and Medical Education Research, Würzburg, Germany; 4 University Hospital Würzburg, Clinic for Dermatology, Venereology and Allergology, Würzburg, Germany

**Keywords:** COVID-19 pandemic, medical students, anxiety, depression, quality of life

## Abstract

**Objectives:**

Changes in academic conditions due to the COVID-19 pandemic are potential stressors for medical students and can make them vulnerable for the development of psychiatric disorders.

Previous pandemics had a negative impairment on well-being due to social isolation and the perceived threat, an increase in fear, anger and frustration and an increase in post-traumatic stress disorder among health professionals. Therefore, this study examines the impact of the COVID-19 pandemic on medical students’ mental health and possible psychological consequences.

**Methods:**

In this anonymous online survey (online 12/01/2021-03/31/2022), we examined the impact of COVID-19 pandemic on mental health of 561 German medical students aged between 18 und 45 years. Perceived anxiety and burden were assessed retrospectively from spring 2020 to autumn 2021. Changes in symptoms of anxiety and depression were assessed using the Hospital Anxiety and Depression Scale (HADS), quality of life was assessed using the WHO Quality of Life Questionnaire (WHOQOL BREF).

**Results:**

Anxiety and burden showed wavelike courses with higher scores in autumn, winter and spring. The scores for depression and anxiety increased after the outbreak of the COVID-19 pandemic compared to the time before (p<.001). Results of a multifactorial ANOVA showed, that previous psychiatric illness (p<.001), being in the first two years of studies (p=.006), higher burden (p=.013) and greater differences in symptoms of depression (p<.001) were associated with a decreased quality of life in medical students.

**Conclusion:**

The COVID-19 pandemic has a negative impact on mental health of medical students and their actual quality of life. Therefore, medical faculties should establish specific support to prevent the development of psychiatric sequelae probably resulting in long-term medical leaves.

## Zusammenfassung

**Zielsetzung:**

Die durch die COVID-19-Pandemie veränderten Studienbedingungen stellen potenzielle Stressoren für Medizinstudierende dar und können sie für die Entwicklung psychiatrischer Erkrankungen anfällig machen.

Frühere Pandemien führten aufgrund der sozialen Isolation und der wahrgenommenen Bedrohung zu negativen Effekten auf das Wohlbefinden, zu einer Zunahme von Angst, Wut und Frustration sowie zu einer Zunahme von posttraumatischen Belastungsstörungen bei Angehörigen der Gesundheitsberufe. In dieser Studie werden daher die Auswirkungen der COVID-19-Pandemie auf die psychische Gesundheit von Medizinstudierenden und mögliche psychologische Folgen untersucht.

**Methodik:**

In dieser anonymen Online-Befragung (online 12/01/2021-03/31/2022) untersuchten wir die Auswirkungen der COVID-19-Pandemie auf die psychische Gesundheit von 561 deutschen Medizinstudierenden im Alter zwischen 18 und 45 Jahren. Die wahrgenommenen Ängste und Belastungen wurden retrospektiv für den Zeitraum Frühjahr 2020 bis Herbst 2021 erhoben. Veränderungen von Angst- und Depressionssymptomen wurden mit der Hospital Anxiety and Depression Scale (HADS) erfasst, die Lebensqualität wurde mit dem WHO Quality of Life Questionnaire (WHOQOL BREF) erfragt.

**Ergebnisse:**

Angst und Belastung zeigten wellenförmige Verläufe mit höheren Werten im Herbst, Winter und Frühjahr. Die Werte für Depressionssymptome und Ängste stiegen nach dem Ausbruch der COVID-19-Pandemie im Vergleich zur Zeit davor an (p<.001). Die Ergebnisse einer multifaktoriellen ANOVA zeigten, dass eine psychiatrische Vorerkrankung (p<.001), die Zugehörigkeit zur Gruppe der Studierenden in den ersten beiden Studienjahren (p=.006), eine höhere Belastung (p=.013) und größere Differenzen zwischen den Depressionssymptomen vor und während der Pandemie (p<.001) mit einer verminderten Lebensqualität bei Medizinstudierenden zusammenhingen.

**Schlussfolgerungen:**

Die COVID-19-Pandemie hat negative Auswirkungen auf die psychische Gesundheit von Medizinstudierenden und ihre aktuelle Lebensqualität. Daher sollten die medizinischen Fakultäten spezifische Unterstützungsangebote einrichten, um die Entwicklung psychiatrischer Folgeerscheinungen bei den angehenden Ärztinnen und Ärzten zu verhindern, die wahrscheinlich zu langfristigen Krankschreibungen führen könnten.

## 1. Introduction

According to the WHO [1], the COVID-19 pandemic caused approximately 514 million infections and 6.24 million deaths worldwide by the end of April 2022. The associated changes in living conditions, as well as infection control measures and the fear of possible infection, represent conceivable stressors that also put health professionals and medical students in a vulnerable position. In addition to varying availability of protective equipment and often increased risk of exposure, the pandemic also had an impact on education for medical students, such as teaching restrictions, virtual lectures and online teaching formats and keeping normally scheduled patient contacts to a minimum [2].

Besides the impact on physical health caused by SARS-CoV-2, the impact on mental health also came to the fore. In this regard various references indicated, younger individuals, students, and women in particular to show higher distress during the pandemic [3]. Due to the pandemic-related restrictions on social life, the reduced opportunities for building social relationships and social exchange and the associated increase in loneliness were identified as possible stress factors in young adulthood [3]. Especially for first-year students, who have to cope with the new challenges of studying as well as a possible change of residence, social interactions are important protective factors. The interruption of face-to-face teaching led to a far-reaching reduction of these. An online survey of students at the Johannes Gutenberg University in Mainz, Germany, conducted before the start of the pandemic (summer 2019) and after the outbreak (June 2020) showed a significant increase in symptoms of depression and loneliness among respondents in the course of the pandemic and higher average symptoms of anxiety and depression among females [3]. According to a cross-sectional study, the COVID-19 pandemic led to greater study-related stress for a majority of students in health-related fields compared to previous semesters [4]. In a further survey on expectations of and stressors for medical students in spring 2020, it was found that students were primarily concerned about their development due to the uncertainty of the situation at that time. In addition to lack of social contacts with teachers and fellow students, reduced opportunities to train practical skills also emerged as stressors [5]. 

In addition, two U.S. studies investigated the impact of the COVID-19 pandemic on mental health of medical students. A large proportion of the students showed symptoms of anxiety (65.9%), with about a third of the participants exceeding the cut-off of a generalized anxiety disorder in terms of the Generalizied Anxiety Disorder Scale-7 (GAD-7). Similar results were found for symptoms of depression, with 56% of the participating students showing depressive symptoms in the Patient Health Questionnaire-9 (PHQ-9) and 24.3% screening positive for the presence of a major depressive disorder. Of particular relevance appears to be the difference in the scores of medical students in the COVID-19 pandemic compared with the general population and medical students without the background of the pandemic. Higher scores for anxiety and depression were demonstrated in women and students in preclinical semesters [6]. Also, in another study in the U.S., 84.1% of students reported anxiety symptoms in spring 2020, when the number of infections and deaths increased sharply in the United States [7].

Impacts on the mental health of healthcare workers were already evident during previous epidemic infection outbreaks [8], [9]. In addition, some affected individuals found it difficult to reconcile their work and the associated increased risk of infection with their role in their families, often leading to feelings of anxiety, anger, and frustration [9]. 

The COVID-19 pandemic has caused severe limits on teaching and students' social life over the last two years of studying, raising concerns about potential long-term negative effects on medical students' mental health and quality of life. Those potential effects and possible risk factors for a deterioration of mental well-being were investigated by means of an anonymous online survey in order to assess the need for preventative actions and support like supervision, coaching or psychological counseling services for medical students. As shown in past analyses, it appears that progress in medical studies may have an impact on student well-being, so this study will also focus on the differences between preclinical semesters (first and second years of study) and clinical semesters (third year and above).

## 2. Methods

### 2.1. Study population and recruitment methods

The survey was conducted at the Mannheim Medical Faculty of the University of Heidelberg and at the Wuerzburg Medical Faculty as an anonymous online survey in which medical students from the first semester onwards, as well as already practicing young physicians could participate. The survey was available online between 12/01/2021 and 03/31/2022. Medical students were recruited at both medical faculties with the support of the deaneries and student departments of the respective universities through social media and public relations. Physicians were reached by the participating clinics and secretariats of the individual specialties through calls for participants via intern mailing lists. Socio-economic circumstances of both cities do not differ (“Dynamic large and medium-sized cities at risk of exclusion”, [10])

For this survey, the software SoSci.Survey (version 3.2.40 SoSci Survey GmbH, Munich, Germany) was used, which allows anonymous data collection without storing the IP address of the participant. Participants were informed about the content, aim and procedure of the survey before taking part in the study and had to actively give their consent to participate in the study.

Medical studies in Germany last approximately twelve semesters or six years. Generally it is divided into two parts, the preclinical section and the clinical section including a practical year at the end. The medical study program in the preclinical semesters in Wuerzburg is a classical study program with topics being taught one after the other, whereas the study program in Mannheim is a model degree program in medicine where the courses are all taught simultaneously in an organ-specific way rather than separately. The preclinical part refers to the first and second year of medical school and is primarily concerned with theoretical principles, whereas the clinical section begins in the third year. Here, in addition to theoretical skills, practical skills are taught as well and insights into everyday clinical life are provided through teaching units on the wards and in direct contact with patients. The practical year comprises the last two semesters of the study program, during which students complete twelve months of practical activities in various clinics. It consists of three tertials at the Wuerzburg faculty and four quarters in Mannheim, during which students work and learn in different specialties. Here, it is mandatory to complete one part in internal medicine and one part in surgery.

The analysis presented here is a partial analysis of the total data set (N=668), which includes only the data of students (N=561) in both the preclinical (N=229) and clinical years of study (N=332). 

Prior to the start of recruitment, there were positive votes from the Ethics Committee II of the Mannheim Medical Faculty of the University of Heidelberg and the Ethics Committee of the University of Wuerzburg (file number MA: 2021-645; WÜ: 2021-120901). The study has been registered with the German Clinical Trials Registry (DRKS-ID: DRKS00028984).

#### 2.2. Survey procedure

**Figure 1:**
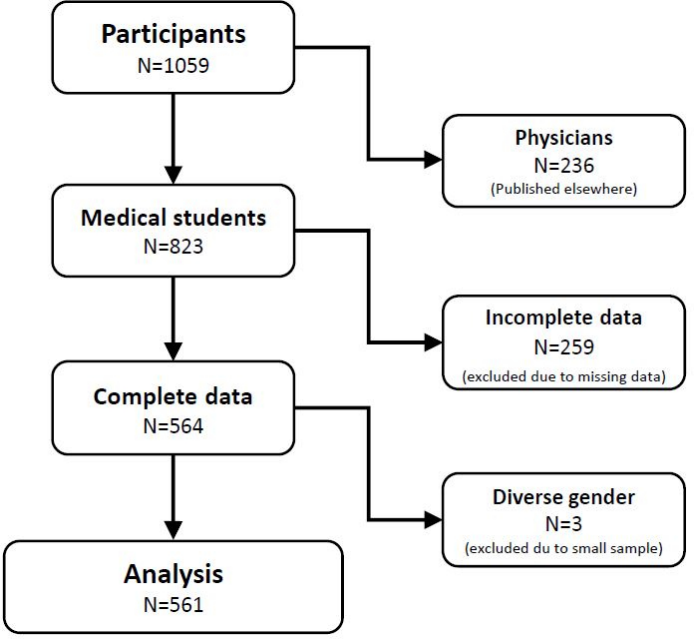
Flowchart of the participants enrolled in the study. Presentation of the exclusion procedure prior the analysis of the presented data.

N=1059 male and female medical students and physicians aged 18 to 45 years participated (for more information see flowchart of enrolled participants, see figure 1 (Fig. 1)) in an online self-assessment questionnaire consisting of existing and validated questionnaires and self-developed questions. The first section of the questionnaire assessed sociodemographic data such as age, gender, marital status and socioeconomic status. The second section asked pandemic-related questions. The focus was on existing protective equipment in hospitals and universities (5-level from “not at all sufficient” to “completely sufficient”), the overall threat of the COVID-19 pandemic to oneself, Germany and the whole world (from “low” via “medium” to “high”) and the effects on family life, social relationships and work/profession (“positive” – “negative” – “neutral”). In the final section we asked questions about mental health before and during the pandemic including a retrospective assessment of subjective anxiety (“How would you rate your subjective anxiety during the pandemic?”, 3-level from “none at all” to “severe”) and subjective burden (“Rate your subjective stress during the pandemic”, 5-level from “none” to “very high”) at 7 measurement time points (spring 2020, summer 2020, fall 2020, winter 2020, spring 2021, summer 2021, fall 2021). Moreover, changes in symptoms of anxiety and depression were assessed on a 4-point scale (from “not at all” to “most of the time”) using the German version of the Hospital Anxiety and Depression Scale (HADS) [11] which were rated by the participants for the period before the outbreak of the pandemic as well as since the outbreak.

In addition participants’ current quality of life was assessed using the German version of the WHO Quality of Life BREF (WHOQOL BREF) [12] including the dimensions of global well-being, physical well-being, psychological well-being, social relationships, and environment. The questions assessing quality of life were recorded on a 5-point scale (from “not at all” to “completely”).

#### 2.3. Statistical analysis

Statistical calculations were performed using IBM SPSS version 27 (IBM Corporation, Armonk, New York). The two-sided significance level was set at =.05 for all tests. Frequency distributions among categories for sociodemographic variables, as well as COVID-19-specific questions about on-site protective equipment, work in COVID-19 care units, and possible psychosocial support in dealing with work, were reported as absolute numbers of cases and percentage frequencies related to the total sample and the two subgroups formed from the total data set (participants from the preclinical and clinical semesters, respectively). 

The significance of differences in subjective anxiety and subjective burden over time from spring 2020 to autumn 2021 between the seven measurement time points was tested using the non-parametric Friedman test. Means and standard deviations were reported for the HADS-A/D and WHOQOL BREF for the total group and the two subgroups. Comparison of the sum scores of the HADS-A/D before and after the onset of the pandemic, respectively, was performed using paired-sample t-tests for both the total sample and subsamples. Differences in mean quality of life scores (WHOQOL BREF) between preclinical vs. clinical semester subsamples were tested for significance using independent samples t-tests. 

A multi-factorial ANOVA was calculated to analyze the influence of the selected variables age, gender, presence of prior mental illness before the pandemic, availability of protection equipment, mean subjective anxiety, mean subjective burden, change in HADS sum score from before to after the outbreak of the pandemic, and year of study on students’ current quality of life.

## 3. Results

### 3.1. Sample description

N=1059 persons participated in the survey, of which N=823 were medical students. The analysis presented here is a partial analysis of the total data set, in which only the data of the medical students were included. The results of the physicians surveyed (N=107 complete data sets) are published elsewhere. In total, complete data sets were available from N=564 students, of which 3 individuals with diverse genders were excluded due to the subsample being too small. Subsequently data of N=561 male and female medical students were included in the analysis. Of these, 229 were in the preclinical semesters of study (1^st^-2^nd^ year) and 332 were in the clinical semesters (3^rd^ year and above). 

Table 1 (Tab. 1) provides an overview of the frequency distribution of sociodemographic variables and COVID-19-specific factors for the total sample and the preclinical and clinical subsamples.

#### 3.2. Subjective anxiety

**Figure 2:**
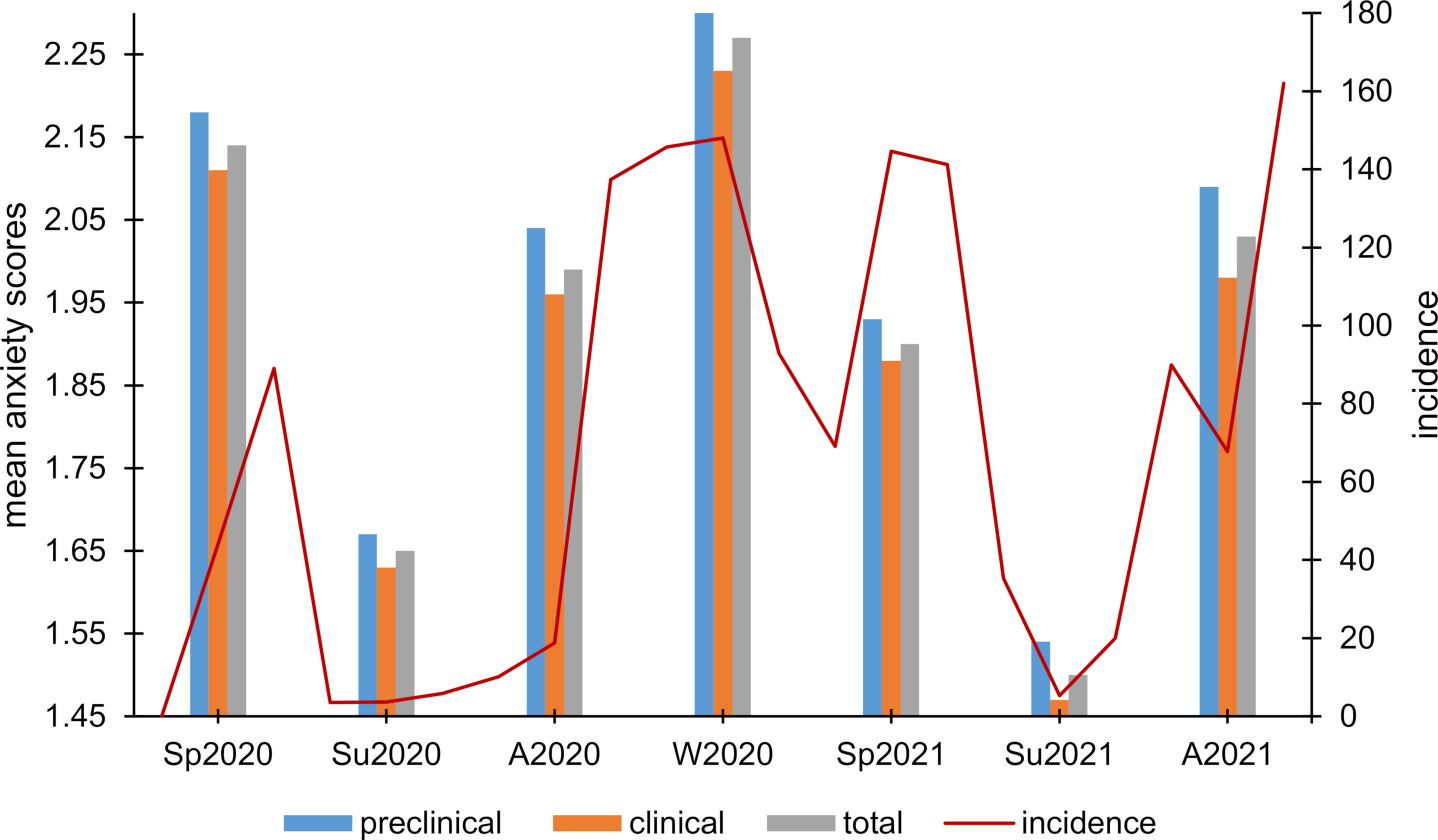
Course of the subjective anxiety. Presentation of mean anxiety scores at the different measurement time points for the total sample and the preclinical and clinical subsamples in relation to the nationwide COVID-19 incidence scores over time [23]

Students' subjective anxiety differed significantly between the seven measurement time points in both the total sample (Friedman test: Chi^2^(6)=666.84, *p*<.001, n=561) and the preclinical (Chi^2^(6)=276.96, *p*<.001, n=229) as well as the clinical (Chi^2^(6)=391.02, *p*<.001, n=332) subsample (see figure 2 (Fig. 2)). A wavelike course was observed, with higher anxiety scores in the autumn, winter, and spring months and lower scores in the summer months, similar to the pattern of COVID-19 incidences.

A detailed table of post-hoc Dunn-Bonferroni tests that test each measurement time point against the other can be found in the online attachment 1 .

#### 3.3. Subjective burden

**Figure 3:**
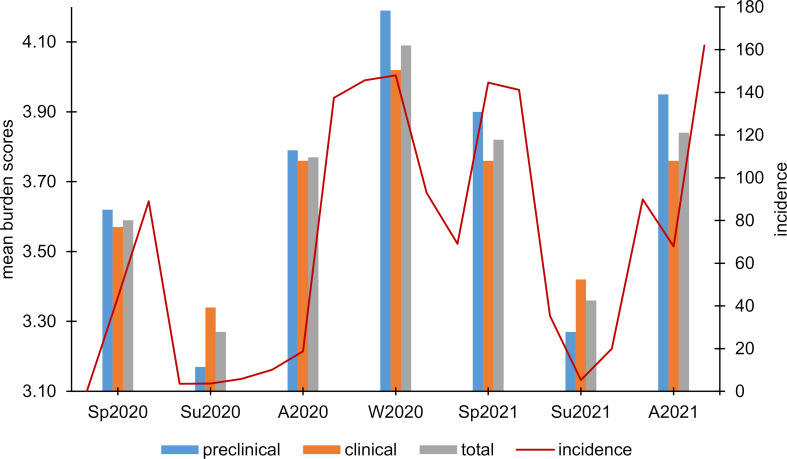
Course of the subjective burden. Presentation of mean burden scores at the different measurement time points for the total sample and the preclinical and clinical subsamples in relation to the nationwide COVID-19 incidence scores over time [23]

A similar wavelike process with lower values in the summer months was shown for subjective burden. This again differed significantly between all seven measurement time points in both the total sample (Friedman test: Chi^2^(6)=474.88, *p*<.001, n=561) and the preclinical (Chi^2^(6)=283.22, *p*<.001, n=229) as well as the clinical (Chi^2^(6)=210.17, *p*<.001, n=332) subsample (see figure 3 (Fig. 3)). A detailed description of the post-hoc Dunn-Bonferroni tests can be found in the online attachment 2 . 

#### 3.4. Change in depression scores

**Figure 4:**
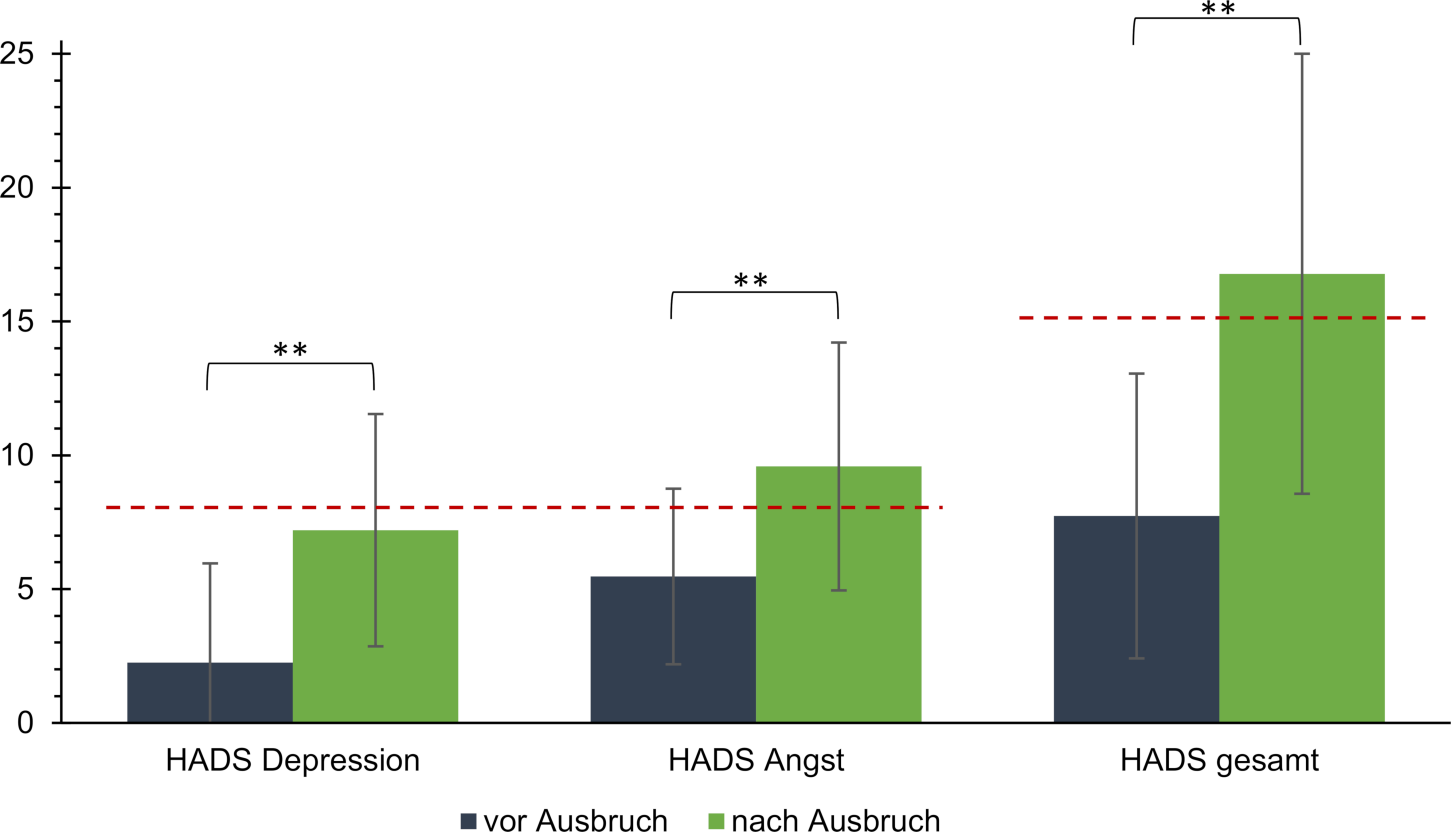
Changes in the HADS Score. Change in sum scores for the HADS total scale and the anxiety and depression subscales before the outbreak of the pandemic compared to after the outbreak of the pandemic. The dashed line corresponds to the cut-offs for a clinically abnormal score.

There was a significant increase in the mean depression scores after the outbreak (ao) of the pandemic compared to the mean depression scores before the outbreak (bo) of the pandemic, both in terms of the total scale of the HADS (bo: *M*=7.73, *SD*=5. 32; ao: *M*=16.78, *SD*=8.22, *t*(560)=-30.74, *p*<.001), as well as the depression subscale (bo: *M*=2.25, *SD*=2.71; ao: *M*=7.20, *SD*=4. 34; *t*(560)=-30.64, *p*P<.001) and the anxiety subscale (bo: *M*=5.47, *SD*=3.28; ao: *M*=9.58, *SD*=4.63, *t*(560)=-26.27, *p*P<.001). It seems particularly relevant that before the outbreak of the pandemic, 11.2% of the students exceeded the cut-off of 15 to a clinically abnormal value; whereas after the outbreak of the pandemic, 56.3% did. A similar picture was seen in the subscales (depression: bo: 5.5%≥8, ao: 41.9%≥8; anxiety: bo: 24.6%≥8, ao: 64.3%≥8), compare figure 4 (Fig. 4).

Looking at the depression scores for the preclinical and clinical subsamples, it can be seen that the differences of the total score of the HADS were significantly higher in the preclinical (*M*=10.24, *SD*=6.78) than in the clinical subsample (*M*=8.24, *SD*=7. 00), *t*(559)=3.372, *p*=.001. The same picture was found in the subscales depression (preclinical: *M*=5.47, *SD*=3.79; clinical: *M*=4.58, *SD*=3.80, *t*(559)=2.727, *p*=.007) and anxiety (preclinical: *M*=4.77, *SD*=3.60; clinical: *M*=3.67, *SD*=3.71, *t*(559)=3.528, *p*<.001).

Consequently, students in the preclinical subsample showed significantly greater increases in depression scores across all dimensions of the HADS during the pandemic than did students in the clinic subsample. No gender differences were observed in terms of increases in HADS scores for the total scale (*t*(559)=1.347, *p*=.179), the depression subscale (*t*(559)=1.525, *p*=.128), or the anxiety subscale (*t*(559)=.962, *p*=.336).

#### 3.5. Subjective quality of life after 2 years of pandemic

Table 2 (Tab. 2) provides an overview of the participants' subjective quality of life in the different domains of the WHOQOL BREF for the total sample and the preclinical as well as the clinical sample, respectively, at the time of the survey after 2 years of pandemic.

The clinical subsample had significantly higher quality of life scores than the preclinical subsample for the global quality of life domain (*t*(559)=-3.88.43, *p*<.001, |d|=. 33), the physical quality of life (*t*(559)=-4.29, *p*<.001, |d|=.37), the psychological quality of life (*t*(559)=-3.59, *p*<.001, |d|=.31), and quality of life related to social relationships (*t*(559)=-3.21, *p*=.001, |d|=.28). The subsamples did not differ in the environmental quality of life (*t*(559)=-1.83, *p*=.069).

#### 3.6. Factors influencing quality of life

The multifactorial ANOVA showed that the presence of a previous mental illness (*F*(1,551)=14.586, *p*<.001, *ηp^2^*=.026), the year of study (*F*(1,551)=7.650, *p*=.006, *ηp^2^*=.014), the mean burden (*F*(1,551)=6.195, *p*=.013, *ηp^2^*=.011) and the difference in depression scores (*F*(1,551)=93.063, *p*<.001, *ηp^2^*=.144) in the total sample have a significant association with or significant main effect on global quality of life. The presence of a previous mental illness (B=-9.790, t(559)=-3.819, *p*<.001), membership in the preclinical subgroup (B=-5.096, *t*(559)=-2.766, *p*=.006) as well as higher burden (B=-4.143, *t*(559)=-2.489, *Pp*=.013) and higher depression scores (B=-1.244, *t*(559)=--9.647, *p*<.001) lead to lower quality of life scores. The variables gender (*p*=.814), age (*p*=.233), presence of protection equipment (*p*=.297), and mean anxiety (*P*=.133) showed no significant association with global quality of life.

It is further shown that the overall model is significant, explaining 26.8% of the variance in global quality of life (*F*(8,551)= 26.532, *p*<.001, adjusted *R^2^*=.268, *n*=560, *ηp^2^*=0.278).

## 4. Discussion

In our survey, similar to the COVID-19 incidence in Germany, subjective anxiety and personal burden each showed wavelike courses with higher burden and anxiety scores in the autumn, winter, and spring months. Apart from current COVID-19 incidence, COVID-19 measures such as restrictions in social life at the relevant time points are also conceivable factors influencing respondents' mental health. For example, restrictions for the general population were less strict in the summer than in the fall and winter months. Furthermore, restrictions for the general population were also particularly high at the beginning of the pandemic in Germany in spring 2020. In addition, a significant increase in depression and anxiety scores could be demonstrated after the outbreak of the pandemic compared to the period before. In contrast to some previous studies that have often identified female gender as a risk factor for lower mental health during the COVID-19 pandemic [13], [14], [15], we did not find gender differences. Rather, preclinical semester students showed significantly higher anxiety and burden scores (except at summer measurement time points) and greater variation over time, as well as significantly greater increases in HADS sum scores than students of the clinical semesters. The results are consistent with a similar study in which first-year medical students had higher stress, anxiety, and depression levels than students in subsequent years of education even before the outbreak of the COVID-19 pandemic [16]. One of the reasons for this could be that first-year students are confronted with significantly more new challenges in their everyday lives. For example, they have to adapt to the university learning environment and at the same time establish a new social environment, often due to relocation. These challenges have been magnified by the pandemic-related restrictions on face-to-face teaching and social contact opportunities. This is supported by our findings on students' current quality of life. Here, preclinical students showed lower global, as well as psychological, physical, and social quality of life.

We were able to show that the presence of a previous mental illness, the year of study, the mean burden and the difference in depression scores had a significant effect on global quality of life. In line with this, a multifactorial ANOVA showed that the presence of a previous mental illness, belonging to the preclinical student group, higher burden and a larger increase in depression scores lead to a lower global quality of life. Consistent with this, previous studies of student mental health during the COVID-19 pandemic have shown high prevalence rates of or increases in anxiety and symptoms of depression [17], [18], [19], as well as reduced well-being [20]. Although these data collections occurred at the beginning of the pandemic and shortly after the introduction of COVID-19 infection protection measures, and thus, unlike our study, no conclusions could yet be drawn about long-term symptom progression and changes in quality of life.

When interpreting our findings, it should be taken into account that this is a retrospective online survey of mental health status. The validity might be limited in terms of the accuracy of the information provided by participants at the seven different past measurement time points due to the fact that we did not specify exact points in time but only time periods and, on the other hand, with such a long period of time, there could be memory errors of the students´ mental health. However, prospective data collection was difficult in the context of the question investigated here, as the course and duration of the COVID-19 pandemic were difficult to be estimated at the onset of the pandemic. Therefore, we decided to use a retrospective survey method. Another conceivable limitation results from the fact that our survey did not record whether participants had ever personally suffered a Covid infection, which could possibly affect mental health. In this regard, a meta-analysis showed that the most common mental health impairments in post-COVID-19 patients were symptoms such as anxiety (range 6.5% to 62%), depression (range 4% to 31%), and post-traumatic stress disorder (range 12.1% to 46.9%) [21].

A further limitation results from the fact that due to the small number of cases of persons with a diverse gender (N=3) and the statistical decision to exclude these persons, only data from two genders (male, female) were included in the analyses. In further studies all genders should be included, if statistical correctness can be ensured by sufficient case numbers. A further limitation results from an existing small overrepresentation of the female gender in our sample compared with the reference collective of all medical students in Germany (gender distribution 2020/2021: 63.18% female; 36.82% male; [22]).

## 5. Conclusions

Despite the mentioned limitations, the present study demonstrates that the COVID-19 pandemic leads to changes in the psychological distress of medical students, with consecutive impact on their quality of life and possible long-term effects on mental health. This suggests the need for the timely establishment of prevention and support services and mechanisms for medical students at the faculties in order to counteract the pandemic-related increased psychological burden of future physicians and to avoid the development of secondary psychiatric diseases and burnout with the risk of long-term disease-related absences.

## Trial registration

The study has been registered at the German Clinical Trials Registry (DRKS-ID: DRKS00028984).

## Authorships

Shared first authorship: Marie Halfmann and Lea Wetzel.

Shared senior authorship: Astrid Schmieder and Anne Koopmann.

## Acknowledgements

MH, LW, AS, AK, FK and SK conceived of the study. MH, LW, NC, AS and AK initiated the study design and implementation of the study. Statistics were carried out by LW, MH, AS and AK. The manuscript was prepared by MH and LW as lead authors as well as FK, SK, AS and AK. All authors contributed to refinement of the study protocol and approved the final manuscript.

## Competing interests

The authors declare that they have no competing interests. 

## Supplementary Material

Pairwise comparisons, post-hoc Dunn-Bonferroni tests; subjective anxiety

Pairwise comparisons, post-hoc Dunn-Bonferroni tests; subjective burden

## Figures and Tables

**Table 1:**
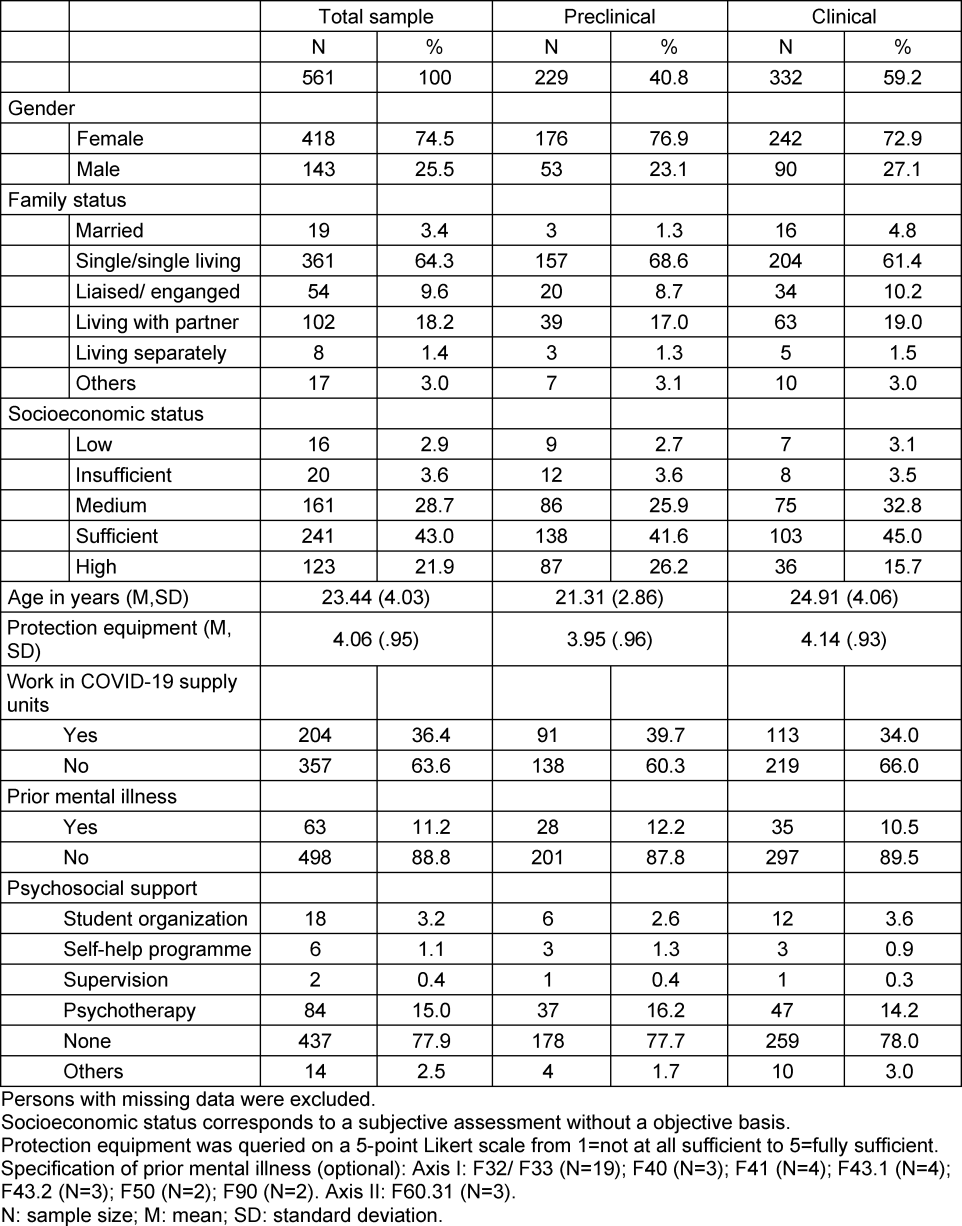
Sociodemographics and COVID-19 situation

**Table 2:**
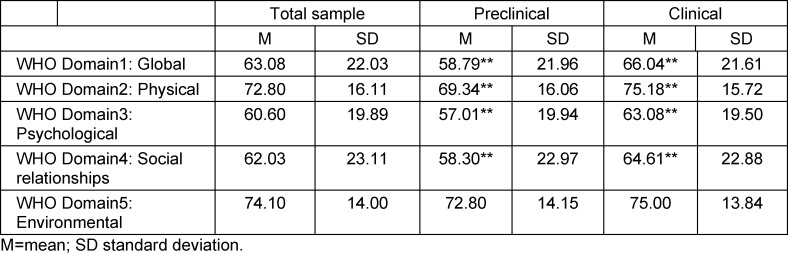
Participants' subjective quality of life after 2 years of pandemic according to WHOQOL BREF
