# Supplementary material for: The impact of COVID-19 pandemic on mental burden and quality of life in medical students – results of an online survey
Source: GMS J Med Educ. 2023 Apr 17;40(2):Doc21. doi: 10.3205/zma001603 (PMC10285365; doi:10.3205/zma001603)
Supplement: Pairwise comparisons, post-hoc Dunn-Bonferroni tests; subjective anxiety [file JME-40-21-s-001.pdf]

Attachment 1: Pairwise comparisons, post-hoc Dunn-Bonferroni tests; subjective anxiety

Sample 1-Sample 2	Test Statistics	Standard Error	Standard Test Statistics	Sig.	Adap. Sig.[a]
Anxiety_Su_2021-Anxiety_Su_2020	,427	,129	3,310	,001	,020
Anxiety_Su_2021-Anxiety_Sp_2021	1,118	,129	8,665	,000	,000
Anxiety_Su_2021-Anxiety_A_2020	1,413	,129	10,952	,000	,000
Anxiety_Su_2021-Anxiety_A_2021	-1,512	,129	-11,726	,000	,000
Anxiety_Su_2021-Anxiety _Sp_2020	1,856	,129	14,386	,000	,000
Anxiety_Su_2021-Anxiety_W_2020	2,228	,129	17,275	,000	,000
Anxiety_Su_2020-Anxiety_Sp_2021	-,691	,129	-5,355	,000	,000
Anxiety_Su_2020-Anxiety_A_2020	-,986	,129	-7,642	,000	,000
Anxiety_Su_2020-Anxiety_A_2021	-1,086	,129	-8,416	,000	,000
Anxiety_Su_2020-Anxiety_Sp_2020	1,429	,129	11,077	,000	,000
Anxiety_Su_2020-Anxiety_W_2020	-1,801	,129	-13,965	,000	,000
Anxiety_Sp_2021-Anxiety_A_2020	,295	,129	2,287	,022	,466
Anxiety_Sp_2021-Anxiety_A_2021	-,395	,129	-3,061	,002	,046
Anxiety_Sp_2021-Anxiety_Sp_2020	,738	,129	5,721	,000	,000
Anxiety_Sp_2021-Anxiety_W_2020	1,111	,129	8,610	,000	,000
Anxiety_A_2020-Anxiety_A_2021	-,100	,129	-,774	,439	1,000
Anxiety_A_2020-Anxiety_Sp_2020	,443	,129	3,434	,001	,012
Anxiety_A_2020-Anxiety_W_2020	-,816	,129	-6,323	,000	,000
Anxiety_A_2021-Anxiety_Sp_2020	,343	,129	2,660	,008	,164

Attachment 1 to Halfmann M, Wetzel L, Castioni N, Kiefer F, König S, Schmieder A, Koopmann A. *The impact of COVID-19 pandemic on mental burden and quality of life in medical students – results of an online survey.* GMS J Med Educ. 2023;40(2):Doc21. DOI: 10.3205/zma001603

Anxiety_A_2021-Anxiety_W_2020	,716	,129	5,549	,000	,000
Anxiety_Sp_2020-Anxiety_W_2020	-,373	,129	-2,888	,004	,081

Each row tests the null hypothesis that the distributions in sample 1 and sample 2 are the same.
Asymptotic significances (two-sided tests) are shown.

a. The significance level is .050.
