# Supplementary material for: The impact of COVID-19 pandemic on mental burden and quality of life in medical students – results of an online survey
Source: GMS J Med Educ. 2023 Apr 17;40(2):Doc21. doi: 10.3205/zma001603 (PMC10285365; doi:10.3205/zma001603)
Supplement: Pairwise comparisons, post-hoc Dunn-Bonferroni tests; subjective burden [file JME-40-21-s-002.pdf]

Attachment 2: Pairwise comparisons, post-hoc Dunn-Bonferroni tests; subjective burden

Sample 1-Sample 2	Test Statistics	Standard Error	Standard Test Statistics	Sig.	Adap. Sig.[a]
Burden_Su_2020-Burden_Su_2021	-,190	,129	-1,472	,141	1,000
Burden_Su_2020-Burden_Sp_2020	,863	,129	6,689	,000	,000
Burden_Su_2020-Burden_A_2020	-1,160	,129	-8,990	,000	,000
Burden_Su_2020-Burden_Sp_2021	-1,220	,129	-9,460	,000	,000
Burden_Su_2020-Burden_A_2021	-1,357	,129	-10,524	,000	,000
Burden_Su_2020-Burden_W_2020	-1,967	,129	-15,250	,000	,000
Burden_Su_2021-Burden_Sp_2020	,673	,129	5,217	,000	,000
Burden_Su_2021-Burden_A_2020	,970	,129	7,518	,000	,000
Burden_Su_2021-Burden_Sp_2021	1,030	,129	7,988	,000	,000
Burden_Su_2021-Burden_A_2021	-1,168	,129	-9,052	,000	,000
Burden_Su_2021-Burden_W_2020	1,777	,129	13,778	,000	,000
Burden_Sp_2020-Burden_A_2020	-,297	,129	-2,301	,021	,449
Burden_Sp_2020-Burden_Sp_2021	-,357	,129	-2,771	,006	,117
Burden_Sp_2020-Burden_A_2021	-,495	,129	-3,835	,000	,003
Burden_Sp_2020-Burden_W_2020	-1,104	,129	-8,561	,000	,000
Burden_A_2020-Burden_Sp_2021	-,061	,129	-,470	,638	1,000
Burden_A_2020-Burden_A_2021	-,198	,129	-1,534	,125	1,000
Burden_A_2020-Burden_W_2020	-,807	,129	-6,260	,000	,000
Burden_Sp_2021-Burden_A_2021	-,137	,129	-1,064	,287	1,000

Attachment 2 to Halfmann M, Wetzel L, Castioni N, Kiefer F, König S, Schmieder A, Koopmann A. *The impact of COVID-19 pandemic on mental burden and quality of life in medical students – results of an online survey.* GMS J Med Educ. 2023;40(2):Doc21. DOI: 10.3205/zma001603

Burden_Sp_2021-Burden_W_2020	,747	,129	5,790	,000	,000
Burden_A_2021-Burden_W_2020	,610	,129	4,726	,000	,000

Each row tests the null hypothesis that the distributions in sample 1 and sample 2 are the same.

Asymptotic significances (two-sided tests) are shown.

a. The significance level is .050.
